# Calcineurin Is a Universal Regulator of Vessel Function—Focus on Vascular Smooth Muscle Cells

**DOI:** 10.3390/cells12182269

**Published:** 2023-09-13

**Authors:** Alexander Nolze, Sebastian Matern, Claudia Grossmann

**Affiliations:** Julius Bernstein Institute of Physiology, Martin Luther University Halle-Wittenberg, 06112 Halle (Saale), Germany

**Keywords:** calcineurin, vascular smooth muscle cells, cardiovascular diseases

## Abstract

Calcineurin, a serine/threonine phosphatase regulating transcription factors like NFaT and CREB, is well known for its immune modulatory effects and role in cardiac hypertrophy. Results from experiments with calcineurin knockout animals and calcineurin inhibitors indicate that calcineurin also plays a crucial role in vascular function, especially in vascular smooth muscle cells (VSMCs). In the aorta, calcineurin stimulates the proliferation and migration of VSMCs in response to vascular injury or angiotensin II administration, leading to pathological vessel wall thickening. In the heart, calcineurin mediates coronary artery formation and VSMC differentiation, which are crucial for proper heart development. In pulmonary VSMCs, calcineurin/NFaT signaling regulates the release of Ca^2+^, resulting in increased vascular tone followed by pulmonary arterial hypertension. In renal VSMCs, calcineurin regulates extracellular matrix secretion promoting fibrosis development. In the mesenteric and cerebral arteries, calcineurin mediates a phenotypic switch of VSMCs leading to altered cell function. Gaining deeper insights into the underlying mechanisms of calcineurin signaling will help researchers to understand developmental and pathogenetical aspects of the vasculature. In this review, we provide an overview of the physiological function and pathophysiology of calcineurin in the vascular system with a focus on vascular smooth muscle cells in different organs. Overall, there are indications that under certain pathological settings reduced calcineurin activity seems to be beneficial for cardiovascular health.

## 1. Introduction

**Figure 1:**
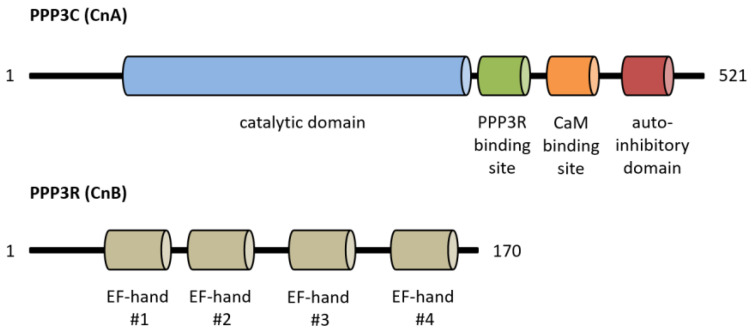
Overview of calcineurin’s structure with important functional protein domains. CaM, calmodulin.

**Figure 2:**
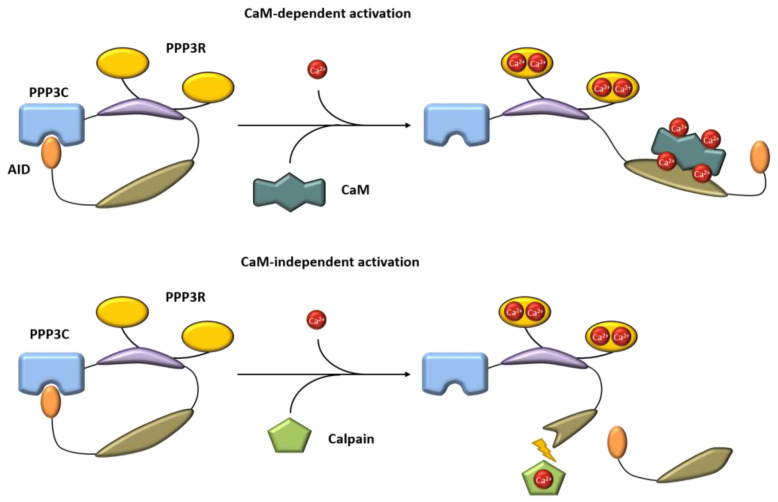
Mechanism of calcineurin activation in a calmodulin-dependent and a calmodulin-independent manner. Blue = catalytic domain, yellow = PPP3R, purple = regulatory domain, dark green = calmodulin binding site. AID, autoinhibitory domain, CaM, calmodulin.

Calcineurin, a Ca^2+^/calmodulin-dependent serine/threonine protein phosphatase, is classified as protein phosphatase 2B (PPP3) [1]. The protein is a heterodimer consisting of a catalytic (PPP3C, ~60 kDa) and a regulatory subunit (PPP3R, ~19 kDa), which are both expressed as multiple isoforms (Figure 1) [2,3]. Three distinct genes, PPP3CA, PPP3CB and PPP3CC, encode the catalytic domain PPP3C. PPP3CA and PPP3CB are ubiquitously expressed and comprise nearly all of the enzymatic activity in most tissues, whereas PPP3CC is mainly expressed in the testis and the brain [4,5,6]. All isoforms of PPP3C encompass a catalytic domain for serine/threonine phosphatase activity and three regulatory domains for auto-inhibitory functions, calmodulin binding and PPP3R binding [7]. Of special interest is the autoinhibitory domain (AID), which binds to the active site of calcineurin in the absence of Ca^2+^/calmodulin, thereby inhibiting the enzymatic function of calcineurin [8]. The regulatory subunit PPP3R, which directs the substrate specificity of the catalytic subunit, is encoded by two genes, PPP3R1 and PPP3R2 [9]. Both possess four EF-hand motifs for Ca^2+^ binding, with the N-terminal Ca^2+^ binding sites having a low Ca^2+^ affinity whereas the C-terminal ones show a high Ca^2+^ binding affinity [7,10]. Full calcineurin activation requires increased intracellular calcium concentrations induced by calcium influx from the extracellular space via different types of calcium channels or by calcium release from intracellular stores, e.g., via activation of the angiotensin II type 1 receptor (AT1R) with angiotensin II (angII) [5,11,12]. Consequently, conformational changes are initiated by Ca^2+^ binding at specific sites in PPP3R, allowing calmodulin binding on the now-accessible respective region of PPP3C, which leads to the dissociation of the auto-inhibitory domain from the active site of PPP3C [10]. This is in accordance with results showing that calcineurin is inactive when calmodulin is not bound to PPP3C because of sterical interference between the autoinhibitory domain and the catalytic site [10]. A second mechanism for calcineurin activation is calmodulin-independent and requires the Ca^2+^-dependent cysteine protease calpain [13]. Calpain can irreversibly activate calcineurin via proteolytic cleavage of the autoinhibitory domain of calcineurin [14]. Overall, there is calmodulin-dependent and calmodulin-independent activation of calcineurin (Figure 2).

After activation, calcineurin dephosphorylates different substrates such as transcription factors, receptors and channels [15,16,17,18]. The best-characterized target of calcineurin is the transcription factor NFaT (nuclear factor of activated T cells). This protein family of transcription factors is composed of five proteins: NFaT1 (NFaTc2), NFaT2 (NFaTc1), NFaT3 (NFaTc4), NFaT4 (NFaTc3) and NFaT5 [19]. Activity of NFaT is mainly regulated via its subcellular localization. In unstimulated cells, inactive phosphorylated NFaT is found in the cytoplasm. Dephosphorylation by activated calcineurin induces a conformational change that exposes the nuclear localization signal of NFaT, leading to nuclear import and transcription of different genes [12,20]. 

NFaTs were first identified in lymphoid cells, and the most prominent function of NFaT is the activation of T cells by the regulation of genes responsible for modulating immune response [15]. Furthermore, NFaT is implicated in the regulation of cell cycle progression, as well as in regulating gene transcription in neurons, skeletal muscle, and the heart [21,22,23], but it also plays an important role in the regulation of physiological functions in the vasculature, namely in vascular smooth muscle cells (VSMC) and endothelial cells [24,25,26]. It is not completely understood what exact role individual NFaT isoforms play in the vasculature, but NFaTc1 (NFaT2) is the most abundant isoform in smooth muscle cells (SMCs) [27]. Strikingly, NFaT has been shown to play an important role in the development of vascular diseases, e.g., in the progression of endothelial dysfunction [28]. In human aortic VSMCs, NFaT activation stimulates cell proliferation and migration by increasing IL-33 expression [29]. Another study describes the effect of calcineurin/NFaT activation on smooth muscle cell proliferation via the calcium-sensing protein STIM1 [30]. Additionally, NFaT plays a major role in the phenotypic regulation of VSMCs [31]. Furthermore, the development of vascular inflammation and remodeling is driven by calcineurin/NFaT signaling, as shown by different studies [32,33,34,35]. Overall, the activation of calcineurin/NFaT is a major factor for the development of vascular diseases.

Another prominent transcription factor regulated by calcineurin is the cyclic AMP-response element (CRE)-binding protein CREB. By dephosphorylation, CREB activity is inhibited, leading to reduced target gene expression [36]. CREB inactivation also influences the expression of glucose-6-phosphate dehydrogenase (G6PDH) followed by reduced reactive oxygen species (ROS) scavenging. An interaction of calcineurin- and cAMP-dependent signaling pathways with the mineralocorticoid receptor (MR) has been shown. The MR, as member of the steroid-receptor family, plays a crucial role in the development of cardiovascular diseases. Aldosterone-induced MR activation increases calcineurin activity mediated via the catalytic beta subunit of calcineurin (PPP3CB) and thereby attenuates CREB phosphorylation [37,38]. 

Not only are NFaT and CREB dephosphorylated by calcineurin, but also the forkhead transcription factors (FOXO), which are involved in metabolic processes, autophagy and cardiomyocyte growth [39,40,41], as well as MEF2 (myocyte-specific enhancer factor 2) and TFEB (transcription factor EB), which both play important roles in regulating skeletal muscle mass and neuronal function [42,43]. 

Besides transcription factors, calcineurin also dephosphorylates receptors and ion channels. Calcineurin regulates synaptic long-term potentiation and depression as well as sympathetic tone by targeting AMPA and NMDA receptors [44,45,46]. Additionally, an interaction between calcineurin and ryanodine receptors has been described, which then regulates Ca^2+^ release [47,48]. Furthermore, calcineurin is implicated in the regulation of the Na^+^/Ca^2+^-exchanger (NCX1) and smooth muscle cell L-type calcium channels [49,50].

Calcineurin activity can be blocked by pharmacological inhibitors that are commonly used as immunosuppressants to avoid allograft rejection or to treat autoimmune diseases. The most famous clinically used inhibitors of calcineurin activity are cyclosporine A (CsA), tacrolimus (FK-506) and pimecrolimus, as well as the more recently developed voclosporine. Mechanistically, these drugs form a complex with immunophilins, including cyclophilin and FK binding proteins, which are endogenous peptidyl-prolyl cis/trans isomerases that contribute to a variety of cellular processes, and inhibit the catalytic activity of calcineurin [51,52]. Both CsA and voclosporine bind to cyclophilin-1, but voclosporine provides a stronger inhibitory effect on calcineurin activity due to a modification in one amino acid residue compared with CsA. Tacrolimus and pimecrolimus both bind to FK binding proteins. The formed protein–protein complexes competitively bind to calcineurin and inhibit NFaT dephosphorylation and activation by blocking the catalytic calcineurin activity itself [53]. Such unspecific blockage of different isoforms of the catalytic subunit as well as the interaction with immunophilins seems to be the reason for many side effects of calcineurin inhibitors, including the development of hypertension due to them influencing tubular salt reabsorption, causing peripheral vasoconstriction and increasing sympathetic tone [54]. Besides proteins inhibiting calcineurin phosphatase activity completely, new inhibitors are available that selectively prevent the interaction between calcineurin and NFaT. There are several docking motifs in NFaT for interaction with calcineurin, and a peptide called VIVIT inhibits protein–protein interaction by mimicking NFaT docking sites [55]. The advantage of VIVIT in comparison with CsA and FK-506 is its more selective inhibition profile, because normal calcineurin function in other targets is not impaired but NFaT activation is interrupted [56].

The AID of calcineurin itself is an endogenous calcineurin activity inhibitor. As mentioned above, with low calcium concentrations the AID blocks enzymatic functions and allows substrate binding after Ca^2+^/Calmodulin binding [9]. In vitro synthesized AID itself inhibits calcineurin in neuronal cells, showing that calcineurin plays a role in excitatory neuronal cell death [57].

Furthermore, cellular proteins regulating calcineurin activity, known as regulators of calcineurin (Rcan1-3), exist, with Rcan1 being the best-studied member of this gene family because Rcan1.4 is part of a feedback loop with calcineurin/NFaT [58]. High Rcan1 levels lead to an inhibition of calcineurin/NFaT signaling in endothelial cells. On the other hand, a complete loss of Rcan1 also impairs calcineurin/NFaT signaling [59,60]. Therefore, low Rcan1 activity is important for proper calcineurin/NFaT signaling. In the late 1990s, another protein called Cabin-1 (calcineurin-binding protein 1, CAIN) was identified as a regulator of calcineurin signaling in T cells [61]. Its inhibiting effect relies on hyperphosphorylation of Cabin-1 by PKC, allowing interaction with calcineurin and blocking NFaT dephosphorylation. Table 1 summarizes calcineurin inhibitors.

## 2. General Calcineurin Function

The classical calcineurin function was initially described in T cells, where calcineurin/NFaT signaling acts as a master regulator of lymphocyte development and the expression of interleukin-2 (IL-2), IL-17 and tumor necrosis factor-α (TNFα) and controls T-cell functions and subsequently immune response [78,79,80,81]. Not only is T-cell activity regulated via calcineurin, but the phosphatase is also expressed in other immune cells, such as B cells, macrophages and dendritic cells [82,83,84]. Hence, repression of calcineurin signaling is a potent target for the suppression of immune response [85]. As calcineurin shows its highest expression in brain tissues, it plays important roles in modulating synaptic plasticity, memory and long-term potentiation [44,86]. Alterations in Ca^2+^ homeostasis due to accumulation of misfolded proteins support the activation of calcineurin in the brain and link calcineurin to pathological processes known from neurodegenerative disorders like Parkinson’s disease [87,88]. Here, clearance of alpha-synuclein aggregates by cathepsin D is disturbed and accompanied by high or low calcineurin activity. On the other hand, moderate calcineurin activity seems to be neuroprotective in neuronal cell culture models [89]. Furthermore, calcineurin plays a role in bone development, and loss of calcineurin leads to osteoporosis [90]. A role in osteoblast differentiation is proposed by Huynh et al., who show that inhibition of calcineurin signaling leads to altered osteoblast differentiation and bone loss [91]. In the gastrointestinal tract, calcineurin signaling is involved in the regulation of secretory processes. In gastric chief cells, calcineurin is responsible for pepsinogen release; in gastric parietal cells, it is responsible for gastric acid secretion [92,93].

Besides in immune cells, the brain, the bone and the gut, calcineurin plays also an important role in the development of cardiovascular diseases, including processes like hypertrophy, inflammation and remodeling. Calcineurin–NFaT signaling is implicated in vascular patterning, myocardial development and heart valve morphogenesis [94,95,96]. Several studies show that calcineurin influences vessel outgrowth. The first remarkable results come from Graef and colleagues, suggesting that calcineurin signaling is necessary for the crosstalk of vessels and the surrounding tissue for proper patterning of the vascular system. The authors demonstrate in a mouse model that inhibition of calcineurin leads to an outgrowth of vessels in regions with high NFaT3 and NFaT4 expression [96]. This seems to be due to the regulation of local vascular endothelial growth factor A (VEGF-A) expression by calcineurin/NFaT signaling. VEGF-A is an important mediator of vasculogenesis and angiogenesis and is essential for vascular development [97]. In conclusion, calcineurin directs the outgrowing vessels into a predefined environment, leading to normal vascular function.

Additionally, the role of calcineurin in heart hypertrophy is well characterized. Mice with reduced PPP3CB activity show a decreased hypertrophic response of the heart upon angII and isoproterenol infusion or pressure overload [98]. On the other hand, a loss of PPP3CB increases the sensitivity of the myocardium to ischemia-reperfusion injury, implying a positive effect of calcineurin signaling in maintenance of myocyte viability [99]. In the kidney, a role for calcineurin/NFaT is well described, as calcineurin inhibitors decrease mortality after transplantation dramatically but lead to chronic nephrotoxicity in the long term, with fibrosis and inflammation in the vessels and glomeruli [85]. A variety of studies show that calcineurin is responsible for the development of glomerular and whole-kidney hypertrophy in diabetic rodents and that calcineurin is activated in diabetes and required for extracellular matrix accumulation in the kidney [100,101]. Experiments with the calcineurin inhibitors FK-506 and CsA lead to the assumption that calcineurin plays an important role in the progression of cardiovascular diseases like hypertension and atherosclerosis [102,103,104]. 

Despite increasing knowledge about calcineurin-dependent mechanisms regulating pathological processes in the cardiovascular system, not everything has been completely understood until now. In this review, we provide an overview of the understanding of the regulation, mechanisms of action and functions of calcineurin in the VSMCs of different organs under physiological and pathological conditions.

### 2.1. Calcineurin in the Aorta

The vessel walls of the aorta and arteries consist of endothelial cells, smooth muscle cells (SMCs) and fibroblasts. Each cell type exerts a distinct role required for the physiological function of the vessel. As an important part of medium- and large-sized arteries, vascular smooth muscle cells (VSMCs) control the regulation of vascular tone. It is known that VSMCs undergo a phenotypic switch under pathological conditions, leading to a loss of their physiological functions and morphology. For example, the cells lose their contractile filaments, leading to a more migratory and proliferative phenotype, which in turn favors vascular remodeling and diseases like hypertension and atherosclerosis [105,106]. Furthermore, VSMCs can sense changes in their environment, such as mechanical stress or hyperlipidemia [107]. 

The role of calcineurin in aortic vascular smooth muscle cells (aVSMCs) comprises the regulation of extracellular matrix secretion, proliferation, migration and inflammation, which are hallmarks of pathological remodeling processes in blood vessels. In the aorta, calcineurin inhibition influences angII-induced neointima and aneurysm formation in mouse models of vascular injury and atherosclerosis [108]. Calcineurin is implicated in angII-induced arterial damage. AngII stimulates aortic intima thickening after vessel injury, whereas CsA administration protects against this hypertrophic response in the aorta. Here, increased angII-mediated aortic vascular smooth muscle cell migration leads to the observed effects [109]. On the other hand, inhibition of calcineurin with CsA stimulates the development of hypertension via not-fully-understood calcineurin-independent mechanisms and possibly by increasing circulating angII levels, which lead to altered renal vascular tone [110,111].

Furthermore, a role of calcineurin in angII-induced hypertension is described in a study by Nieves-Cintrón et al. [112]. Administration of angII increases PKCα-dependent Ca^2+^ sparklets in aVSMCs, whereas a PKCα knockout shows no response to angII regarding Ca^2+^ influx. Additionally, long-term angII infusion in WT mice leads to an elevation of blood pressure with no effect in PKCα KO mice. Furthermore, calcineurin activity is increased in WT mice but not in PKCα KO mice, suggesting that PKCα-dependent Ca^2+^ sparklets are necessary for calcineurin/NFaT activation and the development of angII-induced hypertension. In a study from our lab, we also focused on the role of calcineurin in angII-induced vascular changes [113]. We also showed, in a mouse model, that long-term angII treatment via osmotic minipumps led to increased systolic blood pressure with hypertension and pathological aortic remodeling, including increased media thickness and higher expression of inflammation markers like Serpine1 or Rcan1. In calcineurin knockout mice, the angII-effect was abolished. Many studies provide insights into the effects downstream of high CTGF levels, but we demonstrated that calcineurin modulates CTGF expression via altered EGFR signaling. In detail, we observed that angII stimulated the EGFR-TGFβ-CTGF signaling cascade via HB-EGF calcineurin dependently leading to increased CTGF and consequently to higher collagen production in aortic vascular smooth muscle cells. Blocking angiotensin II type 1 receptor with losartan abolished the effects, whereas AT2R inhibition had no effect. Our results suggest a crucial role for calcineurin in angII-mediated pathological vessel remodeling and that calcineurin KO has protective effects on the vasculature.

In former work, Min et al. proposed a similar role for angII and AT1R in calcineurin signaling in terms of vascular senescence. They investigated whether ATRAP, a receptor-interacting protein inhibiting AT1R function, is able to prevent VSMC senescence [114]. ATRAP is expressed in various tissues and interacts with the carboxyl-terminal domain of the AT1R, whereby the receptor is internalized and AT1R signaling is antagonized. AngII-dependent activation of NFaT transcriptional activity led to an increase in p53 and p21 expression and to a higher number of senescent vascular smooth muscle cells. Aortic vascular smooth muscle cells of ATRAP transgenic mice showed decreased proliferation and senescence after angII stimulation. Furthermore, calcineurin signaling and NFaT activation were reduced in these cells compared with ATRAP wild-type cells. Treatment of wild-type cells with angII and CsA also attenuated proliferation and senescence. In summary, the authors showed that ATRAP is able to inhibit the calcineurin/NFaT signaling pathway to prevent vascular senescence and that calcineurin plays an important role in the pathogenesis of cardiovascular diseases.

In accordance with these findings, several studies are dealing with the role of calcineurin–NFaT signaling in aortic vascular smooth muscle cell proliferation [115,116,117,118]. A role for calcineurin in alpha-adrenergic-receptor-activated aVSMC proliferation has been reported. Treatment of aVSMCs with phenylephrine (PE) increased smooth muscle cell proliferation and cell count. After application of CsA, PE-induced aVSMC growth was diminished [117,118]. Subsequently, NFaTc1 was exclusively located in the nucleus 24 h after PE treatment, and the transcriptional activity of NFaT was increased as shown by luciferase reporter gene assays. CsA was able to attenuate these effects, suggesting that calcineurin activation is necessary for PE-induced aVSMC growth [118]. A study by Li et al. proposes that nitric oxide (NO) inhibits phenylephrine-induced aVSMC proliferation through modulation of intracellular Ca^2+^ concentration and calcineurin activity. Accordingly, after application of CsA, aVSMC proliferation was inhibited and could no longer be influenced by NO donator S-Nitroso-N-acetyl-DL-penicillamin (SNAP) or phenylephrine. [117]. Conversely, the neuropeptide catestatin, an endogenous nicotinic cholinergic antagonist, has a positive proliferative effect on aVSMCs from rat aorta. It increases intracellular calcium and thereby promotes calcineurin/NFaT signaling. Subsequently, transcription of proliferative genes was induced, including cyclin A and c-myc. Administration of CsA abolished these effects, indicating that activation of calcineurin/NFaT signaling is necessary for catestatin-induced smooth muscle cell proliferation [115]. Increased migration and proliferation of vascular smooth muscle cells after vascular injury are a problem in the development of vascular in-stent restenosis in arterial tissue. In a study by Giordano et al., similar to with TGFβ administration, treatment with the calcineurin inhibitor FK-506 stimulated the proliferation and extracellular matrix production of vascular smooth muscle cells [119]. Additionally, inhibition of TGFβ type 1 receptor kinase attenuated FK-506-induced VSMC proliferation. Taken together, this study implies that FK-506 exerts a proliferative effect on VSMCs and is a potential stimulus for neointima formation, although FK-506 reduces calcineurin (and cytokine)-dependent VSMC migration and proliferation [119]. This suggests a calcineurin-independent effect via TGFβ signaling.

Grzesk et al. found that CsA increases calcium influx from extracellular calcium stores, thereby stimulating vascular smooth muscle cell contractility, whereas FK-506 did not show this effect, suggesting that calcineurin regulates smooth muscle cell behavior in different manners. CsA directly stimulates protein kinase C, which leads to L-type calcium channel phosphorylation and induces calcium influx [120]. In a study by Potier et al., it was found that SOCE (store-operated calcium entry) influences the proliferation, migration and apoptosis of aVSMCs [121]. Another remarkable study showed that rapamycin (sirolimus) is able to prevent calcineurin activation by blocking Orai1-mediated Ca^2+^ entry into the cell, thereby inhibiting cell proliferation in VSMCs [122]. The authors showed that another indirect way of blocking Ca^2+^ entry into the cell can also inhibit calcineurin function. Sirolimus prevented SOCE into the cell and thereby activation of NFaT via calcineurin. Additionally, the phosphorylation of the transcription factor CREB was blocked by mTOR inhibition. Overall, these findings suggest that SOCE is an important mechanism for calcineurin activation in VSMCs, which can be inhibited by sirolimus.

Important mediators of remodeling processes include inflammatory events occurring in the vascular wall. It was shown that hyperglycemia induces increased calcineurin/NFaT signaling in vascular smooth muscle cells followed by elevated mRNA and protein expression of the pro-inflammatory cytokine osteopontin (OPN) in resistance arteries and large conduit arteries. Again, inhibition of calcineurin/NFaT signaling with CsA or the NFaT inhibitor A-285222 led to a reversal of increased glucose-induced OPN expression. It was suggested that calcineurin/NFaT acts as a molecular sensor in the vascular wall for the development of vascular damage with high importance for vascular dysfunction [123].

A role for calcineurin/NFaT in matrix-driven inflammatory gene expression was suggested in a publication from Orr et al. Here, aVSMCs plated on a matrix consisting of Col1 exhibited increased VCAM1 gene and protein expression, which can modulate the local inflammatory response within an atherosclerotic plaque. Inhibition of calcineurin by CsA significantly decreased VCAM1 expression. Furthermore, the authors showed that VCAM1 expression in aVSMCs is regulated by NFaT, as administration of CsA or the NFaT inhibitor A-285222 completely blocked VCAM1 expression in aVSMCs plated on a Col1 matrix [124]. Satonaka et al. found an influence of calcineurin on the expression of monocyte chemoattractant protein-1 (MCP-1) in aVSMCs. Usage of a constitutively active mutant of calcineurin promoted MCP-1 expression on transcriptional and protein level, whereas MCP-1 expression was abolished by CsA treatment. Additionally, CsA inhibited MCP-1 expression in the femoral arteries after mechanical injury as well as macrophage infiltration, suggesting a stimulating effect of calcineurin on vascular inflammation [103].

Dysfunction of the vessel is a hallmark of vascular diseases like atherosclerosis or hypertension. Regulating the vascular tone and stiffness of the vessels is important to maintain their physiological condition and blood pressure. Calcineurin plays a role in these processes via Rcan1. Rcan1 is described as an inhibitor of calcineurin activity, but there are also reports showing that Rcan1 can stimulate phosphatase activity [60,125]. Regarding the inhibitory function of Rcan1, in a study by García-Redondo, Rcan1 regulated vascular contractility and stiffness via COX-2 [126]. Rcan1 deficiency led to increased COX-2 expression in aVSMCs and a higher phenylephrine-induced vascular response in myography experiments. When cells were treated with CsA, this increased response was attenuated. In summary, Rcan1 acts here as a negative regulator of calcineurin activity, maintaining physiological vessel contractility and stiffness. In another study, a new role of calcineurin/NFaT signaling in VSMCs for regulating the vascular tone was proposed. There, calcineurin was implicated in PKA-dependent modulation of voltage-gated potassium channels (K_V_). Activation of PKA increased the K_V_ current, whereas inhibition of calcineurin resulted in a decrease in the channel current [127]. Additionally, activation of calcineurin/NFaT was shown to influence large-conductance calcium-activated potassium channels (BK), which are important for the modulation of excitability and the contractile state of SMCs [128]. The authors showed that angII administration activated calcineurin/NFaT signaling and thereby decreased the expression of a subunit of the BK channel, which is responsible for its Ca^2+^ sensitivity. Consequently, proper function of the potassium channel was disrupted, which contributed to the development of hypertension and vascular dysfunction [128]. A role of calcineurin in regulating vascular stiffness was proposed in a study by Valisno et al., where knockout of the transcription factor BCL11B in vascular smooth muscle cells in a mouse model led to increased calcineurin expression and stiffness in aortae from these animals. Here, calcineurin expression and activity were directly correlated with the regulation of vascular tone. As a mechanism for these observations, calcineurin was shown to modulate the phosphorylation of VASP (vasodilator-stimulated phosphoprotein), where decreased VASP phosphorylation increased vascular stiffness [129].

**Figure 3:**
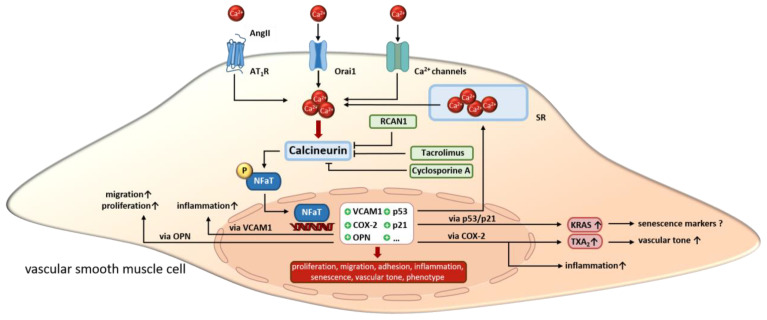
In VSMCs, calcineurin is activated by increasing calcium concentrations resulting from extracellular influx or release from intracellular stores. Upon activation, calcineurin promotes the transcriptional activity of NFaT by dephosphorylation. Altered gene expression regulates VSMC proliferation, migration and inflammation as well as cell phenotype, senescence and vascular tone. Overall, calcineurin inhibitors like cyclosporine A, tacrolimus and endogenous Rcan1 disrupt the signaling cascade but induce side effects. SR, sarcoplasmatic reticulum. “arrow up” indicates an increase; “?” indicates, that the effect is unclear so far.

In summary, the effects of calcineurin in aVSMCs are very diverse and interconnected. A large number of studies suggest an important role for vasoactive substances such as phenylephrine and angII in regulating calcineurin activity in aVSMCs. Through this, the proliferation and migration of aVSMCs is enhanced. On the other hand, SMC senescence is promoted via angII/calcineurin under certain circumstances. Furthermore, calcineurin alters ion channel expression and SOCE, which then modulates Ca^2+^ sensitivity/homeostasis and consequently changes the contractile functions of aVSMCs. Additionally, calcineurin activation promotes inflammation via the upregulating of VCAM or OPN. The effects described above in aVSMCs are all mediated via NFaT (Figure 3). Next, we highlight the functions of calcineurin in SMCs in the heart vasculature.

### 2.2. Calcineurin in the Coronary Arteries

In the coronary arteries, calcineurin plays an important role in the regulation of vessel wall development, inflammatory processes and proliferation. A study revealed that inhibition of calcineurin/NFaT signaling had a negative impact on the vascularization of the developing heart. Treatment of mice embryos with CsA led to disrupted formation of the endothelial tubes, which were fused and restricted to the atrioventricular junction compared with a normally developed network in untreated controls. The same phenotype could be observed in mice embryos with an endothelial-cell-specific deletion of calcineurin [130]. In a report from Yang et al., it was stated that calcineurin–NFaT signaling organizes coronary arterial wall development within a distinct developmental window [131]. Furthermore, calcineurin has been implicated in the development of inflammation in the cardiac vasculature [132,133].

SMCs play an important role in the function of the heart; e.g., they regulate cardiac perfusion by mediating vascular tone [131,134]. It has been shown that alterations in SMCs are responsible for the development of pathological processes, e.g., atherosclerosis or hypertension. SMCs develop from epicardially derived mesenchymal cells and differentiate into SMCs when associating with endothelial cells.

It was demonstrated in a study that calcineurin/NFaT signaling in epicardial progenitor cells directs coronary arterial wall formation [131]. Thereby, calcineurin–NFaT signaling regulates Smad2 expression, which is responsible for TGFβ signaling. Using an epicardial PPP3R1 null mouse model, the authors found a similar phenotype as in Alk5-null hearts (TGFβ1 receptor), with a strongly decreased smooth muscle cell number and reduced maturation [131].

In the previously mentioned study by Zeini et al., impaired endothelial assembly in early coronary vascular development is initiated by the loss or inhibition of calcineurin in endothelial cells, showing that calcineurin is required for proper development of the coronary arteries [130]. Furthermore, activation of calcineurin–NFaT signaling in endothelial cells governs mesenchymal stem cell differentiation into SMCs. Crosstalk between endothelial and mesenchymal stem cells/smooth muscle cells seem to be crucial for coronary artery formation [131]. A study dealing with the clinical aspects of calcineurin/NFaT and its role in VSMC proliferation has shown that FK-506 is able to prevent restenosis in coronary arteries after stent implantation by inhibiting VSMC proliferation. FK-506-treated VSMCs in a cell culture model exhibited a lower increase in cell number compared with controls. Additionally, protein expression of calcineurin, NFaT, and IL-2 was significantly decreased, and knockdown of calcineurin had the same effect [135]. Therefore, the authors claimed that calcineurin is an important molecular target for preventing restenosis in coronary arteries after stent implantation. Another study showed the inhibitory effect of FK-506 on K_V_ channel currents in coronary vascular smooth muscle cells. In comparison with other studies, the observed effects seemed to be independent of calcineurin because the reduced K_V_ currents after FK-506 application occurred much faster than calcineurin inhibition. This observation might explain some side effects of FK-506 as a calcineurin inhibitor [136].

In summary, in coronary arteries, calcineurin influences vessel wall formation during heart development and promotes restenosis after stent implantation as well as atherosclerosis and hypertension. Just like in the aorta, calcineurin promotes the proliferation of VSMCs and stimulates TGFβ signaling. The described effects are mainly mediated by NFaT. The next part will focus on the role of calcineurin in the pulmonary arteries.

### 2.3. Calcineurin in the Pulmonary Arteries

Calcineurin plays an important role in the remodeling processes of the pulmonary arteries, especially in pulmonary arterial hypertension (PAH), which is characterized by increased pulmonary resistance and arterial pressure [137,138]. Furthermore, calcineurin regulates the proliferation and migration of pulmonary artery smooth muscle cells (PASMCs) and modulates Ca^2+^ entry into cells by influencing ion channel activity or expression. Vascular remodeling is often accompanied by endothelial dysfunction, increased migration or proliferation of PASMCs, fibroblast proliferation and abnormal deposition of the extracellular matrix. In PASMCs, calcineurin was shown to induce processes like migration, proliferation, vasoconstriction, cellular hypertrophy and apoptosis [139]. To find a curative approach against PAH, a great effort is being made to identify the underlying molecular mechanisms for these changes in PASMC.

In an article by He et al., it was shown that calcineurin activity is increased after induction of PAH in rat PASMCs, suggesting activated calcineurin/NFaT signaling in PAH. Furthermore, PASMC proliferation and migration are significantly decreased after treatment with CsA. These results indicate that calcineurin seems to be relevant for the development of PAH [139] and that, in PASMCs, key events include the induction of migration and proliferation. A more detailed understanding comes from studies dealing with the regulation of intracellular Ca^2+^ concentrations, as this is an important mediator of vasoconstriction as well as proliferation and migration in PASMCs [121,140,141].

Another study deals with the function of calcineurin in sphingosine-1-phosphate (S1P) and osteopontin-mediated cell proliferation and migration. PASMCs were treated with S1P, leading to increased store-operated calcium entry (SOCE) through activation of PLC followed by augmented calcineurin activity with NFaTc3 dephosphorylation and stimulation of osteopontin gene expression. Osteopontin (OPN) is not only known for its role in bone metabolism but also has functions in cardiac remodeling and pulmonary hypertension [142,143]. Consequently, with BrdU-ELISA, stimulation in PASMC proliferation was detectable. Inhibition of calcineurin with CsA abolished the observed effects. Likewise, old mice displayed higher lung osteopontin levels, right ventricular systolic pressure, and pulmonary vessel muscularization. These changes could be abrogated in osteopontin −/− mice, which also developed attenuated PAH during hypoxia. Accordingly, PASMCs from old mice had a faster growth rate, which could be suppressed by osteopontin antibodies. Taken together, these studies provide new insights into the regulation of PASMC proliferation by calcineurin [144].

Another role of calcineurin/NFaT signaling in vascular remodeling was found by de Frutos et al., who also showed that NFaTc3 is expressed in PASMCs and is activated by chronic hypoxia in a calcineurin-dependent manner. There, inhibition of calcineurin with CsA abolished the NFaTc3-induced upregulation of α-smooth muscle actin (α-SMA) in PASMCs [145]. As thickening of the aortic media is one of the most prominent features of vascular remodeling induced by PASMC hypertrophy, increased expression of α-SMA regulated by calcineurin could be one cause of vascular remodeling.

Regulation of Ca^2+^ entry into the cell is a critical step in the ability of smooth muscle cells to contract, and deregulation can lead to vasoconstriction and pathological remodeling processes. Calcineurin activity is Ca^2+^-dependent, but activated calcineurin can also cause ion channel remodeling with altered calcium influx. As experiments with acid-sensing ion channel 1 (ASIC1) KO mice reveal, Ca^2+^ influx through ASIC1 contributes to PAH induced by chronic hypoxia and endothelin-1. This process is mediated by calcineurin and NFaTc3 nuclear import and requires PICK1 as a scaffolding protein [146]. ASIC1 can also contribute to SOCE and is regulated by calcineurin. It was shown that dephosphorylation of ASIC1 by calcineurin reduces SOCE in PASMCs and that inhibition of calcineurin abolished this effect [146]. However, there are controversial findings regarding the role of calcineurin in regulating SOCE [147]. In a very recent study, Masson et al. provide a possible therapeutic option for treatment of PAH. Here, inhibition of the previously mentioned Orai channels (see Section 2.1 Calcineurin in the Aorta) leads to reduced SOCE, which finally reduces Calcineurin/NFaT activity and abolishes PASMC proliferation and migration [148].

However, not only Ca^2+^ influx via Orai channels, but also influx via TRP channels, can influence calcineurin activity in a pathological manner. In a study by Parpaite et al., chronic hypoxia leads to structural and functional changes in PASMCs, including modulation of ion channel expression. They demonstrated that hypoxia influences transient receptor potential channels’ TRPV4 activity and stimulates TRPV1- and TRPV4-mediated migratory behavior in rat PASMCs. In this process, calcineurin/NFaTc4 signaling seems to be involved, as application of CsA abolished NFaTc4 translocation to the nucleus in response to TRPV1 or TRPV4 activation [149].

Additionally, Li et al. found an influence of calcineurin/NFaT signaling on transient receptor potential channel 6 (TRPC6) expression, which can contribute to pathological proliferation of PASMCs in pulmonary hypertension. They observed an endothelin-1 (ET-1)-mediated upregulation of phosphodiesterase 5 (PDE5) that increased calcineurin/NFaTc4 activity in PASMCs. ET-1 decreased the phosphorylation of NFaTc4 and supported its translocation to the nucleus followed by elevated levels of TRPC6 expression, as TRPC6 was shown to be a direct NFaTc4 target [140]. Furthermore, after TRPC activation by lipopolysaccharide (LPS), TRPC3 and TRPC4 were significantly upregulated in PASMCs. Again, activation of calcineurin was responsible for increased TRPC gene transcription [150]. Another indication of the involvement of calcineurin in PASMC proliferation comes from studies examining the role of serotonin (5-HT) in TRPC channel expression. Nuclear translocation of dephosphorylated NFaT was increased by administration of 5-HT and led to elevated TRPC gene transcription followed by higher PASMC proliferation via increased Ca^2+^ levels. Here, calcineurin stimulated TRPC expression and cell proliferation in a positive manner [151].

Calcineurin not only plays important roles in the regulation of smooth muscle cell proliferation and migration but also mediates inflammation, which can cause vascular remodeling leading to pathological changes in vessel morphology [152]. In a study by Liu et al., calcineurin promoted the inflammatory response of PASMCs. Here, it was postulated that TNF-α has an influence on calcineurin/NFaT signaling. Inhibition of calcineurin/NFaT signaling by administration of mesenchymal-stem-cell-conditioned media suppressed the inflammation-associated overproliferation of pulmonary artery smooth muscle cells [153]. Accordingly, in a rat monocrotaline-induced PAH model, TNF-α in the lung tissue and the plasma increased, as well as calcineurin and NFaTc2 expression in the pulmonary arteries. Transplantation of mesenchymal stem cells reversed the TNF-α and calcineurin/NFaT increase and attenuated PAH, presumably by attenuating proliferation of PASMCs [154].

The regulation of calcineurin/NFaT in PASMCs was also investigated in a study by Yaghi and Sims [155]. Administration of the vasoconstrictor phenylephrine (PE) to PASMCs elevated intracellular Ca^2+^ remarkably and induced translocation of NFaT into the nucleus. With calcineurin inhibitors, the PE-induced intracellular Ca^2+^ increase and NFaT translocation were abolished. Furthermore, the authors showed that rho kinase exerts an important role in calcineurin/NFaT activation. Usage of the rho-kinase blocker Y-27632 abolished translocation of NFaT to the nucleus. Overall, they found that vasoconstrictors not only cause alterations in vascular tone but also regulate gene expression by modulating calcineurin/NFaT activity.

In PASMCs, hypoxia and vasoactive substances like ET-1 and 5-HT induce calcineurin/NFaT-dependent effects, resulting in increased contractile protein expression or ion channel remodeling, which in consequence leads to increased cell migration, proliferation and vasoconstriction, hallmarks of PAH. Additionally, apoptosis was also described. Next, we will provide a short overview of calcineurin in vascular smooth muscle cells in kidney vessels.

### 2.4. Calcineurin in Kidney Vessels

The role of calcineurin in kidney allograft rejection is well described, but a more precise understanding of its cell-type-specific role, especially in smooth muscle cells, is of great interest. Unfortunately, for renal smooth muscle cells, just a few studies exist. Calcineurin exerts important functions in regulating the contraction ability of renal vascular smooth muscle cells and consequently renal blood flow. Therefore, there are indications that renal vascular smooth muscle cells promote the pathogenesis of CsA nephrotoxicity. Usage of calcineurin inhibitors (CNIs) after renal transplantation is still very common, although, e.g., CsA causes nephrotoxicity with hypertension and vascular injury, which primarily affects arterioles with smooth muscle cell vacuolization, loss of definition of cell boundaries, apoptosis and necrosis [156,157,158]. Increased deposits of CD61 (integrin β3)-marked platelets are found in the arteriolar walls of patients treated with calcineurin inhibitors. CD61 is part of the blood coagulation cascade and is used to identify activated platelets, suggesting that calcineurin inhibitors cause vascular injury [157]. Histological changes are similar between renal allografts and native kidneys and between different calcineurin inhibitors [159,160,161,162].

Renal hemodynamic alterations have been identified as another factor contributing to CsA-induced nephrotoxicity. In rats, vasoconstriction of renal afferent arterioles, and, in patients with kidney allografts, a decrease in renal blood flow, has been reported after receipt of CsA [163,164]. Amador et al. showed that the MR in VSMCs, but not in endothelial cells, is involved in hemodynamic alterations induced by CsA and leading to CsA-induced nephrotoxicity. They found that the application of CsA in renal MR KO VSMCs attenuates the plasma urea and creatinine increase typical after CsA administration [165]. On a functional level, CsA-induced phosphorylation of VSMC contractile proteins was prevented in MR KO cells, leading to an attenuated increase in renal vascular resistance. As a mechanism for this observation, attenuation of the renal vascular activity of the L-type- Ca^2+^ channel was found. Furthermore, a connection between calcineurin and the mineralocorticoid receptor (MR) was found in a study that demonstrated that MR-induced inhibition of cAMP/CRE signaling was calcineurin-dependent. Increased calcineurin activity was promoted by activated MR and led to a dephosphorylation of CREB, which in turn attenuated CREB-induced glucose-6-phosphate dehydrogenase expression [166]. NFaT signaling was activated by aldosterone/MR and induced a change in subcellular distribution of the catalytic beta subunit of calcineurin from the cytosol to the nucleus and decreased expression of the endogenous calcineurin inhibitor CAIN [38]. Activation of the glucocorticoid receptor led to a different outcome. SiRNA experiments indicate that PPP3CB and not PPP3CA mediate the observed effects.

In other studies dealing with CsA and renal damage, altered gene and protein expression is also found in smooth muscle cells. For example, FCS-induced COX-2 expression and prostacyclin production were reduced in renal allograft patients after CsA [167]. A role for calcineurin in renal upregulation of the vasoconstrictor endothelin-1 was described by Frank et al., where, after renal transplantation, the expression of endothelin-1 was found to be increased and the usage of ET-1 inhibitors decreased allograft rejection [168]. The authors documented higher ET-1 expression in different tissues of the human kidney, and also in renal vascular smooth muscle cells. Interestingly, ET-1 expression was not altered in kidney grafts, with CsA toxicity implicating an influence of calcineurin on ET-1 expression.

Several studies show a beneficial effect of calcineurin inhibitors for preventing glomerular hypertrophy and matrix accumulation in early diabetic nephropathy through their NFaT inhibitory effect in the glomeruli [100,169]. Conversely, TGFβ was found to regulate ECM via induction of calcium influx and calcineurin in mesangial cells, which then supports glomerular hyperplasia [170]. In early diabetes, blood flow to the glomerulus of the kidney is increased due to inappropriate dilation of the afferent arterioles. This enhanced blood flow is related to a decreased intracellular Ca^2+^ rise and is considered to also facilitate glomerular hypertrophy and shear-induced vessel damage and glomerulosclerosis in late diabetes [171,172,173]. Overall, this suggests that calcineurin may not only play a role in the pathogenesis of diabetic nephropathy in mesangial cells but maybe also in vascular smooth muscle cells that regulate glomerular blood flow.

Additional studies on the function of calcineurin in the kidneys originated from experiments with myofibroblasts. This cell type develops from fibroblasts that have transformed to a smooth-muscle-cell-like phenotype with similar properties to SMCs [174]. It can be shown that inhibition of calcineurin activity with the endogenous calcineurin inhibitor Rcan1 reduces extracellular matrix deposition by myofibroblasts. Additionally, overexpression of Rcan1.4 increases the expression of apoptosis-related proteins. Treatment of the cells with CsA amplifies the effects, suggesting a prominent role for calcineurin in the development of renal fibrosis [175]. In a very recent article, Ume et al. provide an explanation of the contribution of myofibroblasts to calcineurin-inhibitor-induced renal fibrosis. They show that application of FK-506 induces an increase in α-smooth muscle actin as well as an upregulation of TGFβ-receptor activation and downstream Smad2/3 signaling. The observed effects are accompanied by fibroblast-to-myofibroblast transition, with increased cell motility and higher collagen IV expression, suggesting a contribution of calcineurin to the phenotypic switching of fibroblasts to a more smooth-muscle-cell-like phenotype [176].

Overall, in kidney, calcineurin regulates vascular tone by mediating Ca^2+^ influx into smooth muscle cells or ET-1 expression. Additionally, calcineurin promotes extracellular matrix production via TGFβ. Interestingly, calcineurin-dependent changes in ROS scavenging in kidney smooth muscle cells are mediated via CREB and not via NFaT. In the last section, we give an overview of the role of calcineurin in the cerebral and mesenterial vasculature.

### 2.5. Calcineurin in the Cerebral and Mesenterial Vasculature

The role of calcineurin in the smooth muscle cells of the cerebral and mesenterial vasculature is not well described. Most studies deal with alterations in intracellular calcium homeostasis mediated by extracellular signals. In intact cerebral arteries from mice, a modest increase in extracellular glucose led to an increase in intracellular Ca^2+^, calcineurin activation and the nuclear accumulation of NFaTc3, which was mediated by the activation of P2Y receptors by UTP and UDP [177]. Simultaneously, glycogen synthase kinase 3 beta and c-Jun N-terminal kinase activity were reduced by high glucose, resulting in increased NFaT activation via reduced nuclear NFaT export. In aortae and portal veins, high glucose led to a similar increase in NFaT activation [177].

Gomez et al. support that NFaT activation in cerebral artery smooth muscle is induced by UTP through an increase in intracellular Ca^2+^ and the activation of calcineurin. They expand these findings to other G_q11_-coupled receptor agonists like angII, endothelin-1 and prostacyclin F2a [178]. They stress that the rise in intracellular Ca^2+^ results from Ca^2+^ release from the sarcoplasmic reticulum through IP3 receptors and Ca^2+^ influx through L-type voltage-dependent Ca^2+^ channels. However, depolarization-induced Ca^2+^ influx failed to induce NFaT translocation to the nucleus. Ca^2+^ sparks released by ryanodine receptors even exerted a negative influence on NFaT activation [178]. In smooth muscle cells from rat cerebral resistance arteries, the Ca^2+^ removal rate after temporary membrane-depolarization-triggered Ca^2+^ currents was dependent on caveolin-1, caveolin-3 and calcineurin [179]. In hyperglycemic mice on a high-fat diet, AKAP150 is required for the activation of calcineurin/NFaT signaling via Ca^2+^ influx through L-type calcium channels. NFaT activation suppresses the expression of a subunit of large-conductance calcium-activated potassium channels (BKCa) and thereby reduces its calcium sensitivity, which increases the vasoconstriction of resistance arteries. These effects were investigated in cerebral and mesenteric arteries and their corresponding VSMCs. In AKAP150 KO mice on a high-fat diet, enhanced vasoconstriction and an increase in systemic blood pressure was absent [180].

The influence of calcineurin on neointima formation in the carotid arteries after balloon injury is controversial. Liu et al. report that calcineurin inhibitors like CsA and GFP-VIVIT can attenuate neointima formation by 40% and reduce the proliferation of smooth muscle cells. Conversely, NFaT leads to the activation of PDGF-BB and COX-2 in cultured aortic vascular smooth muscle cells [181]. In contrast, Waller et al. report no beneficial effect and even an aggravating effect of CsA on intima hyperplasia in a rat carotid artery balloon injury model [182]. In intracranial aneurysm, a VSMC phenotypic switch is reported that is mediated by increased TRPC6, calcineurin and NFaT expression that leads to enhanced NOX4, p22phox and p47phox expression and ROS production followed by the progression of intracranial aneurysm [183].

Another easily available vessel used to analyze the effect of calcineurin and its inhibitors is the mesenteric arteries. Here, the activation of potassium channels led to hyperpolarization and vasodilation. In rat mesenteric vascular smooth muscle cells, the activity of voltage-gated potassium channels was upheld caveolae-dependently by tonic PKA activation, which could be reversed by calcineurin, thus indicating a mechanism by which calcineurin favors vasoconstriction [127].

In VSMCs from the mesenteric arteries, Na^+^-HCO_3_^−^ cotransporter NBCn1 interacts with the catalytic calcineurin beta subunit. Furthermore, intracellular Ca^2+^ activates NBCn1 in a calcineurin-dependent way and protects cells against intracellular acidification [184]. Calcineurin inhibitors, on the other hand, augment the intracellular acidification of VSMCs, for example during norepinephrine-induced artery contractions.

In the small mesenteric arteries and aortae, experiments with KO mice of the endogenous calcineurin inhibitor Rcan1 suggest that, through inhibition of calcineurin and the NF-KB pathway, Rcan1 inhibits COX-2 expression and activity to maintain normal vascular contractility and stiffness [126]. Accordingly, deficiency in Rcan1 led to an increase in phenylephrine-induced vasoconstrictor response and thromboxane A2 levels. Inhibition of COX-2 by CsA was previously reported by Jespersen et al. in human aortic VSMCs [167]. Others have also found an attenuated vasoconstrictor response in Rcan1 KO mice in response to phenylephrine compared with WT mice. Overall contractility was not affected; only sensitivity to agonists was decreased. Furthermore, NO synthase inhibitors potentiated vasoconstriction in KO animals, suggesting that elevated NO production is involved in the reduced vasoconstriction [185]. In endothelial cells, Rcan1 is furthermore known as a regulator of angiogenesis.

Further results indicating that CsA may exert its nephrotoxic and hypertensive side effect by acting as a vasoconstrictor have come from experiments with rat mesenteric arteries, where CsA induces increased vasoconstriction in response to agonists like noradrenaline and vasopressin. Increased calcium influx in VSMCs upon treatment with vasoconstrictors like angII, serotonin and endothelin-1 was found to be a possible mechanism. However, loading of intracellular calcium pools and interactions with cyclophilins and calcineurin were not involved [186].

Overall, in the smooth muscle cells of the cerebral and mesenterial vasculature, calcineurin regulates intracellular calcium homeostasis, thereby promoting vasoconstriction. These effects are initiated by high glucose, UTP, angII and ET-1, which increase calcium release from intracellular stores and promote calcineurin/NFaT activity. Additionally, the calcineurin/NFaT-dependent upregulation of genes like COX-2 and PDGF-BB, which stimulate smooth muscle cell proliferation, is described.

## 3. Conclusions

In summary, calcineurin plays an essential role in the regulation of physiological and pathophysiological smooth muscle cell function in the vasculature. Dysregulation of this well balanced signaling pathway, which modulates smooth muscle cell proliferation, migration and differentiation has severe consequences. Although calcineurin inhibitors lead to reduced allograft rejection after organ transplantation, they can cause pathological outcomes like hypertension, inflammation and endothelial dysfunction in the vasculature, suggesting positive as well as negative effects of calcineurin. As a putative reason for the observed effects, altered Ca^2+^ homeostasis modulated by calcineurin in VSMCs was found. An increase in intracellular calcium concentration is driven either by release from the sarcoplasmatic reticulum or by entry via ion channels from the extracellular space. Calcium then leads to calcineurin/NFaT activation and is also modulated by calcineurin/NFaT itself, e.g., via ASIC1. Furthermore, calcineurin/NFaT signaling directly promotes the expression of genes which are responsible for inflammatory events (OPN, VCAM, MCP-1) or proliferation (TRPC, CTGF). One role of calcineurin/CREB is described in terms of ROS scavenging. Nevertheless, the best-investigated calcineurin signaling pathway in VSMCs involves NFaT.

Much progress has been made in recent years to elucidate calcineurin signaling events with the identification of downstream targets of this signaling cascade, but most of the responsible downstream signaling events are not completely understood so far, especially in VSMCs. To evaluate these, further experiments should be conducted with calcineurin-knockout cell cultures and in vivo models to avoid the side effects of calcineurin inhibitors. Additionally, other known calcineurin targets like FOXO and MEF2 need to be investigated further in vascular smooth muscle cells.

Understanding the calcineurin signaling pathways causing these pathological effects in the smooth muscle cells of the vasculature will help to improve the treatment of diseases like pulmonary arterial hypertension and chronic kidney disease.

## Figures and Tables

**Table 1:** Summary of known calcineurin inhibitors.

Name of Inhibitor	Source	References
Cyclosporine A (CsA)	exogenous	[62,63,64,65]
Tacrolimus (FK-506)	exogenous	[66,67,68]
Pimecrolimus	exogenous	[69,70]
Voclosporine	exogenous	[71,72]
VIVIT	exogenous	[55,56,73]
Rcan1	endogenous	[74,75]
Cabin-1 (CAIN)	endogenous	[61,76]
AID (autoinhibitory domain)	endogenous	[77]

## Data Availability

No new data were created or analyzed in this study. Data sharing is not applicable to this article.
